# Influenza A (H1N1) virus infection and TNF-308, IL6, and IL8 polymorphisms in Egyptian population: a case–control study

**DOI:** 10.1186/s41936-019-0131-1

**Published:** 2019-11-21

**Authors:** Shaimaa Moustafa Elsayed, Omayma Mohamed Hassanein, Nagwa Hassan Ali Hassan

**Affiliations:** 1 Molecular Biology & Epigenetics, Faculty of Medicine, Medical Ain Shams Research institute (MASRI), Ain Shams University Hospitals, Cairo, Egypt; 2 Molecular Biology Department, Faculty of Medicine, Ain Shams Research Institute (MASRI), Ain Shams University Hospitals, Cairo, Egypt; 3 Department of Zoology, Faculty of Science, Ain Shams University, Cairo, Egypt

**Keywords:** Influenza, Cytokine polymorphisms, TNF-308, IL6 and IL8

## Abstract

**Background:**

The importance of influenza is increasing mainly because of the appearance of novel pandemic strains such as swine and avian. Each year, influenza has spread around the world causing about 250,000–500,000 deaths and more than 5 million cases of severe illness.

The objective is as follows: evaluating the outcomes of patients with influenza A (H1N1) virus in relation to certain TNF-308, IL6, and IL8 polymorphisms and identifying the associated factors with the severe outcome.

**Subject and methods:**

This is a case–control study. The cases were patients confirmed by real-time polymerase chain reaction (RT-PCR) to be influenza A (H1N1) virus infected. The controls were healthy individuals. Medical history and outcome of the disease was registered. In all study participants, polymorphisms of TNF rs1800629, IL6 rs18138879, and IL8 rs4073; odds ratio (OR); and the 95% confidence interval (95% CI) were calculated.

**Results:**

Infection with influenza A (H1N1) virus was associated more with the following genotypes: TNF-308 AA (OR = 4.041; 95% CI = 1.215–13.4) and IL8 AA (OR = 3.273; 95% CI = 1.372–7.805). According to our study results, HCV (OR = 3.2, 95% CI 1.2–8.5), renal disease (OR = 3.4, 95% CI 0.9–13.6), cancer (OR = 3.1, 95% CI 0.3–31.1), TB (OR = 8.4, 95% CI 1.8–39.7), ICU (OR = 2.9, 95%1.2–7.1), and mortality (OR = 7.9, 95% CI 0.9–67.4) are considered as risk factors for influenza A (H1N1)-infected patients.

**Conclusions:**

Our findings concluded that TNF-308 (AA) and IL8 (AA) polymorphisms may increase the susceptibility to be infected with H1N1influenza virus.

## Background

Influenza is an acute and contagious viral respiratory infection that affects 5–10% of adults and 20–30% of children, reaching 290,000–650,000 deaths annually worldwide (Iuliano, Roguski, & Chang et al., 2018).

There are three types of influenza viruses: types A and B cause epidemic disease in humans on an annual basis, while type C causes only sporadic disease. Type A influenza viruses are further subtyped based on differences in the surface glycoproteins hemagglutinin (HA) and neuraminidase (NA). Sixteen subtypes of HA and nine of NA circulate in wild aquatic birds (Lexau et al., 2005).

To identify risk factors for severe disease, and hence to determine the optimal case management and prevention, more clinical and treatment data of influenza A (H1N1) virus are needed.

The secretion of cytokines by infected cells appears to be necessary for the initiation of the immunological response that controls the replication of the virus (Michaelis, Doerr, & Cinatl, 2009). In addition, the presence of immunopathological mechanisms, such as hypercytokinemia (“cytokine storm”), generally is considered to contribute to the severest evolution of the infection (Mainers et al., 2008; Michaelis et al., 2009).

Elevated levels of pro-inflammatory cytokines and chemokines (e.g., TNFα, IFNγ, IL-1, IL-6, IL-8, IL-9, IL-12 IL-15, and IL-17) have been found, up to 10 days after the onset of symptoms, in the plasma of patients with acute respiratory distress syndrome (ARDS) caused by influenza A/H1N1 (Bermejo-Martin et al., 2009; To et al., 2010; Hagau et al., 2010)*.*

The genes that code for these molecules are polymorphic, and certain alleles have been associated with susceptibility to various diseases that cover a wide range of pathologies, from infectious to oncological, including pulmonary and systemic diseases (Paskulin et al., 2011; Wang et al., 2010).

The role of polymorphism of the genes encoding these cytokines in the severity of the disease is not clear. The extensive polymorphism of these molecules may be associated with the high mortality rate during the 2009 influenza A/H1N1 pandemic in Mexico. That genetic factor of the host may influence the nature and intensity of the inflammatory immune response (Morales-García et al., 2012).

## Materials and methods

We aimed to detect the relationship between polymorphism of tumor necrosis factor-308 (TNF-308), interleukin 6 (IL6), and interleukin 8 (IL8) and severity of patients infected with influenza A (H1N1) virus.The nasopharyngeal swab and blood specimens used in this study were received from the Clinics of Medicine, Surgery and Gynecology Departments, Ain Shams University Hospitals, from November 2014 to May 2017.The study was reviewed and approved by the Faculty of Medicine Ain Shams University Ethical Committee. The specimens were collected from 200 patients who were suspected to be infected with influenza virus (influenza-like illness (ILI)). The nasopharyngeal swabs were immediately kept at 4 °C in viral transport media (VTM) consisting of HBSS (Hanks’ Balanced Salt Solution 1X), HEPES (4-(2-hydroxyethyl)-1-piperazineethanesulfonic acid 1M), CaCl2, MgCl_2_, Pen Strep (Pencilin Steptomycin), and albumin from bovine serum. Specimen were immediately transported to the Molecular Biology Research Unit laboratories in Medical Ain Shams Research Institute (MASRI) as fast as possible for molecular examination by real-time polymorphism chain reaction (RT-PCR) according to CDC (2009) protocol to confirm or exclude infection by H1N1 influenza besides sub-typing which were done for H1N1-positive cases.

For high cost of reagents of amplification, 50 patients positive for H1N1virus (group I) and 50 patients negative for H1N1virus (influenza-like illness (ILI)) (group II) were randomly chosen. To detect polymorphisms of TNF-308, IL6, and IL8, RT-PCR amplification was used.

Genomic DNA was extracted from blood samples coated with EDTA from group I and group II. Another 50 blood samples were collected from healthy individuals to be used as control cases (group III).

Severity index was defined by assigning 1 point to each of the following: use of supplemental oxygen, duration of hospital stay ≥ 5days, partial pressure of oxygen (PaO_2_), and admission to an intensive care unit and mortality, based on variables reported in previous studies (Martinello, Chen, Weibel, & Kahn, 2002; Gilca et al., 2006).

Written informed consent, demographic characteristics, clinical history, and radiographic data were collected for all patients from patient’s sheets.

### SNPs typing

Genomic DNA was isolated from the blood using a DNA extraction kit following the manufacturer’s standard protocol (QIAGEN diagnostic, USA).

Genotyping of DNA samples was done for TNF-308 (rs1800629), IL6 (rs1818879), and IL8 (rs4073) polymorphisms using TaqMan commercial probes (Applied Biosystems, USA).

The protocol for RT-PCR was as follows: 15 ng DNA, 15 μL of TaqMan universal PCR master mix, and 6.5 μL of each probe:

- TNF (rs1800629): GAGGCAATAGGTTTTGAGGGGCATG [A/G] GGACGGGGTTCAGC CTCCAGGGTCC.
- IL6 (rs1818879): AGACGAGCTGGGCGCAGTGGCTCAC [A/G] CCTATAATCCCAGC ACTTTGGGAGG
- IL8 (rs4073): TTATCTAGAAATAAAAAAGCATACA [A/T] TTGATAATTCACCAAAT TGTGGAGC.

The amplification recommended by Morales-García et al. (2012) was as follows: 94 °C (3 min), 61 °C (1 min), and 72 °C (1 min), followed by 35 cycles of 94 °C (1 min), 61 °C (1 min), and 72 °C (1 min,), and a final cycle of 94 °C (1 min),61 °C (1 min), and 72 °C (5 min).

### Statistical analysis

Data were analyzed using Statistical Program for Social Science (SPSS) version 20.0 (SPSS, Chicago, IL, USA). Quantitative data were expressed as mean ± standard deviation (SD). Qualitative data were expressed as frequency and percentage. An independent-samples *t* test of significance was used to compare between two sample means. A one-way analysis of variance (ANOVA) was used to compare means of two or more sample means. Chi-square (*χ*^2^) test of significance was used in order to compare proportions between two qualitative parameters. Binary logistic regression was used to predict the outcome of categorical variable based on one or more predictor variables.

The confidence interval was set to 95%, and the margin of error was set to 5%. So, the *p* value was considered significant as the following: probability (*p* value) < 0.05 was considered significant and *p* value > 0.05 was considered insignificant.

## Results

The patients were separated into positive influenza A (H1N1) virus (group I) and negative influenza A (H1N1) virus (group II) besides healthy individuals to be used as control (group III).

In group I, 33 (66%) of patients infected with positive influenza A (H1N1) were males and 17 (34%) were females with a mean age of 44.14 ± 7.33 years (Table 1). In group II, 22 (44%) were males and 28 (56%) were females with a mean age of 44.54 ± 7.12 years. In group III, 19 (38%) were males and 31(62%) were females with a mean age of 37.22 ± 6.33 years. Typical signs and symptoms of flu-like illness such as fever, sore throat, and cough were significantly more common among group I. Also, vomiting was significantly more common among group I. HCV was higher for patients in group I than for those in group II (34% vs.14%, respectively, *p* = 0.19).

**Table 1:** Demographical and clinical characteristics of group I, group II, and group III

Parameters	Group I (*n* = 50)	Group II (*n* = 50)	Group III (*n* = 50)	*p* value
Age (years, mean ± SD)	44.14 ± 7.33	44.54 ± 7.33	37.22 ± 6.33	0.001*
Sex (male/female%)	(66%/34%)	(44%/56%)	(38%/62%)	0.013*
HCV	17 (34.0%)	7 (14.0%)		0.019*
Renal disease	9 (18.0%)	3 (6.0%)		0.065
Respiratory disease	20 (40.0%)	8 (16.0%)		0.265
CVD	10 (20.0%)	11 (22.0%)		0.806
Hypertension	9 (18.0%)	11 (22.0%)		0.617
Cancer	3 (6.0%)	1 (2.0%)		0.307
Diabetes	40 (80.0%)	15 (30.0%)		0.624
TB	13 (26.0%)	2 (4.0%)		0.976
Smoking history	11 (22.0%)	19 (38.0%)		0.081
PaO_2_	39 (78.0%)	27 (54.0%)		0.686
Mortality	7 (14.0%)	1 (2.0%)		0.027*
ICU	40 (80.0%)	29 (58.0%)		0.017*
Chest X-ray				
Infiltrate	7 (14%)	19 (38.0%)		0.012*
Consolidation	30 (60%)	14 (28.0%)		0.003*
Chest infection	8 (16%)	11 (22.0%)		0.601
Others	5 (10%)	6 (12.0%)		0.976
Fever	48 (96%)	30 (60%)		0.00*
Cough	39 (78%)	19 (38.0%)		0.004*
Sputum	50 (100.0%)	50 (100.0%)		1.000
Sore throat	45 (90)	34 (68)		0.007*
Headache	35 (70)	42 (84)		0.096
Dyspnea	10 (20)	5 (10)		0.161
Diarrhea	15 (30)	10 (18.5)		0.248
Vomiting	10 (20)	2 (0.04)		0.0138

*SD* standard deviation, *CVD* cardiac vascular disease, *PaO*_*2*_ partial pressure of oxygen, ICU intensive care unit, *HCV* hepatitis C virus

*p values are significant

The mortality was higher for patients in group I than for those in group II (14% vs. 2%, respectively, *p* = 0.027). We observed severity of the disease (PaO_2_ < 60mmHg) in 78% of patients in group I and in 54% in group II, although there was no statistically significant difference (*p* = 0.68). Eighty percent of patients in group I warranted admission to the intensive care unit (ICU), a statistically significant difference found when compared with group II (*p* = 0.017). Sixty percent of patients in group I had radiological signs of pulmonary compromise, statistically significant when compared with group II (*p* = 0.03).

Regression analysis of clinical data in group I and group II showed risk association with disease susceptibility and infection by A (H1N1) (Table 2).

**Table 2:** Risk of influenza A/H1N1 infection in relation to clinical data

Clinical data	Group I vs. group II		
	*χ*^2^	*p* value	Odds ratio (95% CI)
HCV	5.5	0.001*	3.2 (1.2–8.5)
Renal disease	3.4	0.032*	3.4 (0.9–13.6)
Respiratory disease	7.1	0.004*	3.5 (1.4–9.0)
CVD	0.1	0.403	0.9 (0.3–2.3)
Hypertension	0.3	0.308	0.8 (0.3–2.1)
Cancer	1.0	0.155	3.1 (0.3–31.1)
Diabetes	25.2	0.0002*	9.3 (3.7–23.4)
TB	9.5	0.001*	8.4 (1.8–39.7)
Smoking history	3.0	0.040*	0.5 (0.2–1.1)
PaO_2_	6.4	0.006*	0.3 (0.1–0.8)
Mortality	4.9	0.014*	7.9 (0.9–67.4)
ICU	5.7	0.009*	2.9 (1.2–7.1)

*p values are significant

Carriers of A allele TNF-308 had an increased risk of becoming infected with A (H1N1) virus (odds ratio, OR = 2.49), and this risk was higher for TNF-308 AA homozygous genotype (OR = 4.04). On the other hand, our findings demonstrated the existence of TNF-308 GG with association to protection (*p* < 0.05, OR ≤ 1.0). IL8A allele showed statistically significant association with infection (odds ratio = 2.2), and IL8 AA homozygous genotype had a statistical significance with an elevated OR of 3.27. In IL6, neither allelic nor genotypic analysis showed any association with infection (Table 3).

**Table 3:** Comparison of genotypic and allelic frequencies of studied genes between group I and group III

Gene	Genotype	Group I vs. group III			
		*p* value	Odds ratio (95% CI)	OR allelic (95% CI)	
TNF308	GG	0.002*	0.28 (0.1–0.69)	A allele 2.49 (1.40–4.44)	G allele 0.40 (0.23–0.71)
	AG	0.21	1.38 (0.63–3.0)		
	AA	0.00*	4.041 (1.215–13.4)		
IL8	TT	0.066	0.4633 (0.17–0 .28)	A allele 2.2 (1.24–.90)	T allele 0.45 (0.26–0.81)
	AT	0.07	0.57 (0.26–1.3)		
	AA	0.00*	3.23 (1.3–7.81)		
IL6	GG	0.41	0.91 (0.39–2.13)	A allele 1.179 (0.6718–2.068)	G allele 0.8485 (0.4837–1.489)
	AG	0.34	0.85 (0.4–1.9)		
	AA	0.20	1.61 (0.5–4.9)		

*p values are significant

## Discussion

We examined polymorphisms in TNF-308, IL-8, and IL-6. These polymorphisms have been previously associated with inflammatory status in various diseases or with mortality following influenza A (H1N1) virus infection (Melk et al., 2003; Patel et al., 2010).

TNF-308 polymorphisms have been shown to be key acute-phase cytokine polymorphisms that are involved in inflammation in several infections. However, the influence of these polymorphisms on cytokine production induced by respiratory viruses is not well described (Tang, Li, Wu, Shyr, & Edwards, 2007).

The present results indicated that the TNF-308 A allele and A/A genotype were associated with a higher risk of infection by influenza A/H1N1, while the homozygous TNF-308G/G genotype had a trend toward being associated with protection from infection. This was in agreement with Joel et al. (2013) who found that the TNF-308 carriers of A allele and AA genotype were associated with susceptibility to influenza infection. By contrast, a study conducted by Antonopoulou, Baziaka, Tsaganos, Raftogiannis, and Koutoukas (2012) found no role of TNF308 in influenza infection. In our results, we found in group I a significant relation between TNF genotypes and diabetes, TB, HCV, renal disease, respiratory disease, cardiovascular disease (CVD), chest X-ray, mortality, and ICU hospitalization and noted an increase of severity illness in patients with TNF-308 AA homozygous genotype carriers.

We found no association between IL6 and infection on either allelic or genotypic analysis. On the other hand, we found a significant association of the IL6 with these clinical diseases (TB, renal disease, respiratory disease, CVD) in group I. This was in agreement with the earlier studies (Joel et al., 2013; Kaiser, Fritz, & Straus, 2001; Shen, Hou, & Chen, 2011). But these were in contrast to study done by Morales-García et al. (2012) who found no association with the IL6 and the clinical disease.

The AA genotype and A allele of the IL-8 is related to patient susceptibility to parenchymal infection and is correlated with the severity of infection in pediatric acute pyelonephritis (Cheng, Lee, Tsau, et al., 2011). Interestingly, the T>A polymorphism has recently been reported to be a risk factor of other lung diseases, including bronchial asthma (Heinzmann, Ahlert, Kurtz, Berner, & Deichmann, 2004) and bronchiolitis caused by respiratory syncytial virus (Hacking et al., 2004; Hull, Thomson, & Kwiatkowski, 2000). It is interesting to mention that, even though we did not find any significant relation between the IL8 polymorphism and mortality, those with IL8A allele or AA homozygous genotype had an increased risk of becoming infected with influenza A (H1N1) virus. Like the study done by Yu et al. (2011), we found some diseases that might determine whether the patient would be infected or have a more aggressive disease. Among them were diabetes, HCV, TB, renal disease, and respiratory disease.

The clinical features of our hospitalized patients infected with influenza A (H1N1) virus (group I) included fever, cough, and sore throat which were generally similar to other reports, whereas the incidence of gastrointestinal symptoms such as vomiting and diarrhea were much lower than those previously reported (BinSaeed, 2010; Cao et al., 2009; Ruef, 2007; Webb et al., 2009).

The eight mortality cases in our study were distributed as follows: one case from group II and seven in group I. We observed that patients who died with a severe clinical course and up to ICU were more in the H3N2 subtype of A (H1N1).

Our study has limitations because of expensive cost of the reagents used and the sample size; however, in spite of the reduced number of patients studied, we were able to observe that single-nucleotide polymorphisms (SNPs) showed significant differences in the distribution of genotypes in terms of infection, mortality, and analytical data between cases and controls, strongly suggesting that they are associated with the disease. The study of these polymorphisms could predict the behavior that the infection will bring during future outbreaks in our country.

In conclusion, it was concluded that TNF-308 (AA) and IL8 (AA) polymorphisms increase the susceptibility to be infected with the influenza A (H1N1) virus. Genetic variants within genes involved in the inflammatory process could have contributed to the differences in clinical behavior of the infection in different parts of the country during the influenza H1N1 outbreak, thus leading to more severe disease and higher mortality.

## Data Availability

The relevant data and materials are available in the present study.

## Abbreviations

ARDS: Acute respiratory distress syndrome
CDC: Center for Disease Control and Prevention
CVD: Cardiovascular disease
HBSS: Hanks’ Balanced Salt Solution 1X
HCV: Hepatitis C virus
HEPES: 4-(2-Hydroxyethyl)-1-piperazineethanesulfonic acid 1M
ICU: Intensive care unit
*IL6*: Interleukin 6
PaO_2_: Partial pressure of oxygen
SNPs: Single-nucleotide polymorphisms
TNF-308: Tumor necrosis factor-308
VTM: Viral transport media
